# Development and testing of a mobile application for periodontal diagnosis

**DOI:** 10.4317/jced.59338

**Published:** 2022-03-01

**Authors:** Luisa-María Sánchez-Otálvaro, Yesid Jiménez-Rivero, Ricardo-Andrés Velasquez, Javier-Enrique Botero

**Affiliations:** 1 Facultad de Odontología, Universidad de Antioquia, Calle 70 No. 52-21, Medellín-Colombia; 2 Sistemas Embebidos e Inteligencia Computacional SISTEMIC, Facultad de Ingeniería. Universidad de Antioquia, Calle 70 No. 52-21, Medellín-Colombia

## Abstract

**Background:**

The new classification of periodontal diseases introduced a new set of rules for periodontal diagnosis. The objective of this study was to develop and test the implementation of a mobile device application for periodontal diagnosis.

**Material and Methods:**

An integral algorithm that included periodontal health / related conditions and periodontitis was developed based on the classification of periodontal diseases of 2018. A mobile application for Android implementing the algorithm was developed using the framework MIT App Inventor. Once the app was debugged for glitches and performance of the algorithm, it was tested with 20 voluntary dental students, postgraduate students of periodontology, and professors in an academic setting. Participants were asked to determine the diagnosis of 10 predetermined clinical cases using two strategies: diagnosis based on knowledge and with the PerioSmart app. The results were tabulated, and the concordance rate was calculated.

**Results:**

In general, the use of the PerioSmart application had a better concordance rate than diagnosis based on knowledge. In particular, the mobile app was better in determining the type of diagnosis, stage/grade of periodontitis, and with better efficiency.

**Conclusions:**

The mobile device application demonstrated efficiency and good concordance rate and therefore can improve the periodontal diagnosis.

** Key words:**Mobile application, periodontal diagnosis, periodontitis, gingivitis.

## Introduction

Periodontal disease starts as the marginal inflammatory reaction of the gingivae termed ¨gingivitis¨. It is the result of the accumulation of microbial biofilm around the gingival margin. However, left untreated, the inflammatory reaction slowly progresses and continues affecting the periodontal ligament, cementum, and alveolar bone leading to the destruction of the tooth-supporting apparatus. This latter stage is termed ¨periodontitis¨ and is an important cause of tooth loss in adults (1,2).

A new classification for periodontal diseases was proposed in 2018 as the joint result of the American Academy of Periodontology and the European Federation of Periodontology. This new system discretized the diagnosis of periodontal health conditions and periodontitis based on objective clinical parameters. One important change was the elimination of the term ¨aggressive periodontitis¨, and the addition of staging and grading of periodontitis, which makes the diagnosis more comprehensive (3,4). However, due to the numerous periodontal conditions and the clinical parameters thresholds, it could be difficult to completely implement it by memory, especially by dental students.

An algorithm is a sequence of logical instructions where there is always an initial state and a final state. Algorithms are characterized in that they are finite and have well-defined inputs and outputs. Today, algorithms can be used to help solve periodontally related problems such as diagnosis and be implemented in mobile applications. Tonetti and Sanz (2019) (5) recommended the use of an algorithm proposed to determine the stage and grade of periodontitis mainly for clinical practice and education. However, to the extent of our knowledge, there is no mobile application that could aid in periodontal diagnosis. Therefore, this study aimed to develop and test the implementation of a mobile device application for periodontal diagnosis.

## Material and Methods

The study protocol was revised by the institutional review board of the Facultad de Odontología (Universidad de Antioquia, Colombia; 85-2021) and was conducted following the Helsinki Declaration of 1975, as revised in 2013.

**Figure 1:**
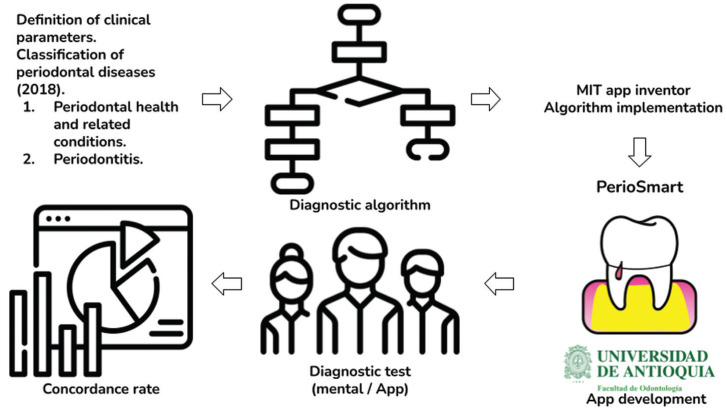
Workflow of the mobile application development and testing.

As the first step for this study (Fig. 1), the diagnostic algorithm was developed using the latest classification of periodontal diseases as a reference (3,4). Each periodontal clinical parameter (probing depth, periodontal attachment level, bleeding on probing, the ratio of bone loss/age) and complimentary parameters such as the number of teeth lost, history of past periodontitis/surgery or treatment, cigarette smoking, and glycemia were clearly defined. Each parameter is used as inputs in the sequence. The first output of the algorithm determines between periodontal health / related conditions and periodontitis. The possible outputs in this stage are intact periodontium, gingivitis in an intact periodontium, health or gingivitis in a reduced periodontium in a non-periodontitis patient, health or gingivitis in a successfully treated periodontitis patient. If the output is periodontitis, then the algorithm determines the stage and grade. The determination of stage and grade were clearly defined following the new classification for periodontal diseases. The final output when periodontitis is determined includes its corresponding stage and grade (e.g.: periodontitis stage III grade B).

After the algorithm was developed and revised, it was systematized for use in mobile devices running Android operating systems (Fig. 1). For this matter, the free MIT App inventor development environment was used (https://appinventor.mit.edu/ Massachusetts Institute of Technology). Each input and output were introduced in the blocks-based coding that resulted in the creation of PerioSmart. First, the application opens with general instructions for the user and there is no need for an internet connection or registration. Then a series of question-like fields presents the user with the options which can be easily introduced or selected. The result is the most probable periodontal diagnosis based on the information introduced. The application does not determine the diagnosis by itself, but rather uses the information introduced by the user. Therefore, this application was developed for the education of dental students and professionals with basic knowledge in periodontology.

The mobile application was debugged and tested for consistent results with 10 predetermined clinical cases by the researchers (LMS, YJR, and JEB). Adjustments were made until the result of the diagnosis was always predetermined. Then, the mobile application was pilot tested by inviting 20 voluntary participants which included dental students, postgraduate students of periodontology, and professors from the Facultad de Odontología of the Universidad de Antioquia. Each participant was asked to determine the diagnosis of 1 or more of the 10 predetermined clinical cases using 2 strategies: mental diagnosis and PerioSmart. Next, the participant filled a Google form that collected information regarding the diagnosis obtained and time spent.

For analysis, data were categorized as: diagnostic strategy (mental diagnosis and PerioSmart), type of periodontal diagnosis (health/related conditions and periodontitis), and severity of periodontitis (stage and grade). The efficiency was calculated as the mean time spent to obtain the diagnosis with each strategy. The data was tabulated and presented as a frequency for categorical variables and mean ± standard deviation (SD) for continuous variables. The concordance rate was calculated for each diagnosis strategy (mental vs. PerioSmart) and graded as: 0.01-0.20 slight, 0.21-0.40 fair, 0.41-0.60 moderate, 0.61-0.80 substantial, and 0.81-1.00 almost perfect or perfect. Differences between continuous variables were established using the student t-test (IBM Corp. Released 2020. IBM SPSS Statistics for Windows, Version 27.0. Armonk, NY: IBM Corp). Statistical differences were assumed when P≤0.05.

## Results

Table 1 shows the concordance rate of mental diagnosis and PerioSmart. In general, while mental diagnosis had a moderate concordance rate (0.52), PerioSmart produced a substantial concordance rate (0.80). Concerning the diagnosis according to periodontal health / related conditions and periodontitis (Table 2), PerioSmart showed an almost perfect concordance rate (0.92) determining periodontitis diagnosis as compared to mental diagnosis (0.44). In the same manner, PerioSmart produced a substantial concordance rate (0.69) when determining health /related conditions in comparison to the moderate concordance rate (0.59) of mental diagnosis.

**Table 1:**
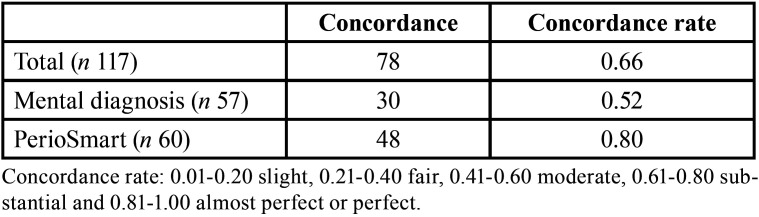
The general concordance rate of mental diagnosis and PerioSmart.

**Table 2:**
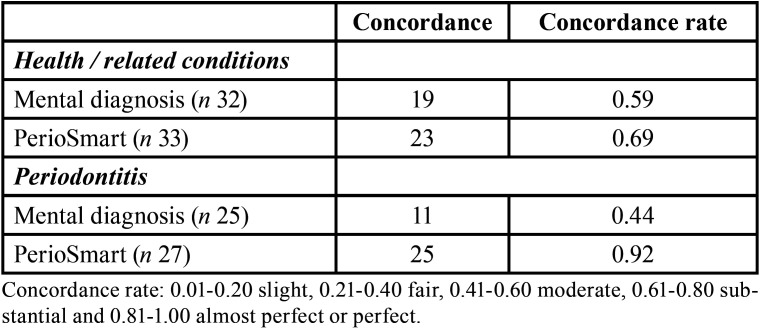
The concordance rate for the determination of the type of periodontal diagnosis.

The determination of stage and grade of periodontitis is depicted in Table 3. PerioSmart had an almost perfect concordance rate for a grade of periodontitis (0.92) as compared to the moderate concordance rate of mental diagnosis (0.44). The determination of the stage of periodontitis showed a substantial concordance rate (0.69) for PerioSmart versus a substantial concordance rate of mental diagnosis (0.59).

**Table 3:**
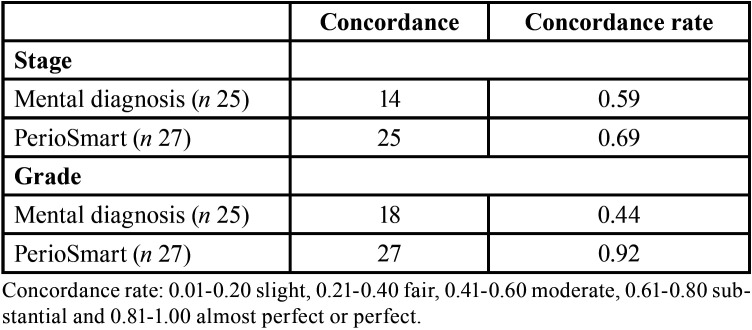
The concordance rate for the determination of the stage and grade of periodontitis.

The efficiency of diagnosis using both strategies is shown in Table 4. PerioSmart was more efficient in determining the periodontal diagnosis (2.08 ± 0.98 minutes) in comparison to mental diagnosis (6.07 ± 3.31 minutes).

**Table 4:**
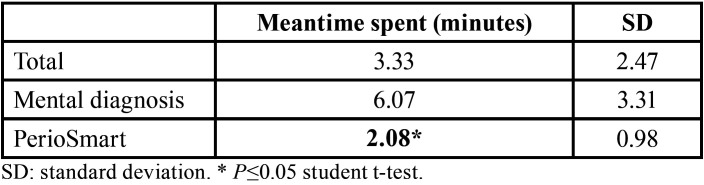
The efficiency of the determination of periodontal diagnosis.

## Discussion

To the best of our knowledge, this is the first study to address the development of mobile applications that could aid in the periodontal diagnosis for educational and professional purposes.

In general, the use of the PerioSmart application for Android devices had a better concordance rate than mental diagnosis. Specifically, the mobile app was better in determining the type of diagnosis, stage/grade of periodontitis and with better efficiency. However, it is important to note that the execution of the diagnosis algorithm of the PerioSmart app is based on the information entered by the user. Therefore, users of the app should have a previous understanding of the periodontal classification and periodontal parameters recorded in the periodontal chart and dental history. For example, according to users, the most challenging part of the diagnosis was determining the stage, which had lower concordance rates than determining periodontitis from health. Depending on the periodontal site that was used as a reference to enter the value for periodontal attachment level, the algorithm established the stage but was not always the same site used to establish the predetermined case resulting in false positive/negative results. In addition, some users were not sure how to apply complexity parameters to advance the stage. This was observed when dental students were invited to test the app. Postgraduate periodontology students and professors had a better and fast acceptance of the app and with resulting better concordance in the diagnosis. Dental students hesitated more when defining the periodontal condition of the predetermined clinical case and therefore, spent more time thinking about the diagnosis. The new classification of periodontal diseases presents numerous diagnoses with different clinical parameter thresholds that more often than not, are hard to memorize and there are still some gray areas that need the critical rationale of the clinician to make the diagnosis. Participants said that when they used the app, the questions were very specific about periodontal parameters that define the periodontal condition and therefore improved their understanding of the new classification system. In contrast, during mental diagnosis, they depended on previous knowledge and had to look up the definition of each periodontal condition. While this would be normal in the learning curve of students, we consider that PerioSmart could be a helpful tool for learning and acquiring the basis of periodontal diagnosis for future practice and help dental students and professionals familiarize themselves with the general rules of the new classification of periodontal diseases.

In recent years, the use of mobile apps has shown that they have the potential to complement and improve the differential diagnosis of diseases and used in academic environments as a complement to the education process (6,7). Ravidà *et al*. (2020) (8) developed a nomogram that efficiently predicted (AUC 0.81) patients at higher risk of tooth loss using the clinical parameters of staging and grading of periodontitis and therefore a reliable application of decision models using the new classification of periodontitis. Unfortunately, there are no studies in the periodontal field using mobile applications and thus it was not feasible to compare our results. However, mobile apps do not replace the scientific rationale and clinical experience of the practitioner. It should be emphasized that the process of critical thinking could be improved by developing available technologies. While this was a preliminary study, it allowed us to observe the efficiency of a simple diagnosis algorithm automatized in a mobile application that in future studies we pretend to upgrade to include machine learning for direct analysis of the periodontal chart. It is our goal to develop a mobile application in multiple languages that would improve the periodontal practice and give the clinician additional diagnosis tools.

## Conclusions

The mobile device application demonstrated efficiency and good concordance rate and therefore can improve the periodontal diagnosis in the dental academic setting.
